# Transvaginal specimen extraction after combined laparoscopic splenectomy and hysterectomy: Introduction to NOSE (Natural Orifice Specimen Extraction) in a community hospital^☆^

**DOI:** 10.1016/j.ijscr.2013.07.039

**Published:** 2013-10-01

**Authors:** Mohan Ramalingam, Jon King, Lisa Jaacks

**Affiliations:** 18250N 32nd Street, Unit 1034, Phoenix, AZ 85032, United States

**Keywords:** Natural orifice surgery, Transvaginal specimen extraction, Splenectomy, Hysterectomy

## Abstract

**INTRODUCTION:**

Developments in the field of minimally invasive surgery have led to interest in NOTES (natural orifice transluminal endoscopic surgery). Even as technologies continue to evolve and develop, interest in some of the advantages of specimen retrieval transvaginally has been roused and we describe a case of combined laparoscopic splenectomy and hysterectomy with transvaginal retrieval of both specimens.

**PRESENTATION OF CASE:**

Patient underwent laparoscopic splenectomy and robot-assisted hysterectomy with transvaginal delivery of specimens. Total operative time was 245 min with no complications. Closure of the colpotomy was achieved laparoscopically. Post-operative course was unremarkable. Patient has done well clinically at 18 months follow-up except for an episode of post-coital spotting, which resolved spontaneously.

**DISCUSSION:**

We explored the technical feasibility of concurrent laparoscopic splenectomy and hysterectomy along with transvaginal retrieval of both solid organs without morcellation. We wanted to illustrate the fact that transvaginal organ extraction may be performed safely in a community or district hospital with standard instruments without incurring additional cost, morbidity or increased operating time.

**CONCLUSION:**

Transvaginal specimen retrieval was technically easy to accomplish. Our patient has not experienced any infectious complications or sexual dysfunction to date. For surgeons exploring an alternative to transabdominal specimen retrieval, transvaginal NOSE is an attractive proposition with several advantages. When combined with a gynecological procedure that involves a colpotomy, this may present a unique opportunity to explore the utility of NOSE.

## 1 Introduction

As we enter the second decade of the 20th century, it heralds promising advances in minimally invasive surgery, one of which is NOTES. As far as cosmesis is concerned, NOTES represents the zenith of scarless surgery. John Hunter prophesized in 1762, that “surgery, gaining much from the general advance of knowledge, will be rendered both knifeless and bloodless”.

In 2004, Kalloo and colleagues^1^ embarked on a series of porcine transoral, transgastric approaches to the peritoneal cavity and in subsequent years, performed a diverse group of surgeries ranging from tubal ligations to cholecystectomies. The first recorded NOTES procedure in humans was a transgastric appendectomy, demonstrated in 2005 by Rao and Reddy via a video presentation and the first published report in the U.S. was on June 25th 2007 by Swanstrom,^2^ who performed a transgastric cholecystectomy. 2005 also independently marked the year by which all surgeries had been performed laparoscopically, a rising testament to the growing popularity and penchant for minimally invasive techniques in surgery.

NOTES has been described as the next paradigm shift in surgery but a number of obstacles still remain. These were eloquently described in the original “white paper” published in 2006^3^ as a collaborative effort between the SAGES and ASGE groups. The technical difficulties related to gaining access to the peritoneal cavity, closure of the viscerotomy, concerns over infection, difficulties in spatial orientation and visualization, control of pneumoperitoneum and development of multi-tasking platforms. Many of these questions remain partially answered at best but it is becoming clear that the choice of access for intra-abdominal procedures in women is through the vagina.

The transvaginal approach is not novel – ventroscopy was described in 1901 by Dimitri Ott, the same year that Georg Kelling independently described laparoscopy. Culdoscopy was described by Decker and Cherry in 1944 and transvaginal appendectomies at the time of hysteroscopy using open instruments was noted by Bueno et al. in 1949.

Open transvaginal hysterectomies have been shown in a Cochrane review article^4^ to be superior to transabdominal hysterectomies with speedier return to normal, fewer febrile episodes or unspecified infections and shorter duration of hospital stay. These advantages were also observed when comparing laparoscopic vaginal hysterectomies versus laparoscopic abdominal hysterectomies in which there were fewer febrile episodes or unspecified infection and shorter operation time.^5,6^

The vaginal route of specimen extraction avoids the morbidity associated with the abdominal incision and feasibility studies have shown it to be applicable for most intra-abdominal organs^7–10^ with minimal added morbidity. Nevertheless there remains a fair amount of skepticism on the part of surgeons and patients to try this new approach. We explore the case of a patient with unrelated but synchronous surgical and gynecological problems wherein the opportunity presented itself for splenic extraction via the colpotomy created for the laparoscopic vaginal hysterectomy.

## 2 Case presentation

Our patient is a 48-year-old female who has a history of localized early stage right renal cell carcinoma and had undergone an open right radical nephrectomy in 2002 via a right flank incision. She is in remission currently.

During her surveillance, an MRI done in August 2010 detected an incidental lesion in her spleen measuring 3.9 cm. A follow up MRI done 3 months later showed the lesion to have increased in size to 4.5 cm. Interventional radiology performed a CT guided biopsy and the pathology showed it to be a possible spindle cell tumor or an inflammatory myofibroblastic tumor. A PET CT done showed low level activity in the splenic lesion. She denied any other local or constitutional symptoms.

She had previously undergone a left oophorectomy and salphingectomy for an ectopic pregnancy, both via a Pfannenstiel incision. She has 3 children, all vaginal delivery.

Due to her menorrhagia for the past couple of months, an ultrasound of her pelvis was done which showed large uterine fibroids. She was scheduled for a laparoscopic hysterectomy in 3 weeks.

Physical examination was unremarkable except for her previous Pfannenstiel and right flank incisions.

Due to the indeterminate nature of her splenic lesion with concerns for malignancy, patient was agreeable to undergoing a splenectomy. Discussion with her gynecologist then yielded the possibility doing both surgeries on the same day, possibly doing the laparoscopic splenectomy first, then the laparoscopic hysterectomy. After a careful review of the literature, we also pursued the idea of transvaginally extracting the spleen after the hysterectomy was performed. Pneumococcal and Hib vaccines were given 2 weeks prior to surgery.

She underwent surgery in February 2011. Pre-operatively, the left ureter was stented to protect the left ureter in view of her single remaining kidney. The laparoscopic splenectomy was conducted with the patient in the supine position. One 12 mm port and 3 other 5 mm ports were placed in a diamond configuration in the left upper quadrant.

The splenectomy was performed with standard laparoscopic dissection techniques. Hem-O-Lok clips (Weck Closure Systems, Research Triangle Park, NC) were applied to the splenic vessels. No complications were encountered.

The remaining attachments were taken down and the spleen was passed into a 15 mm Endo Catch bag (Covidien, Dublin, Ireland). The splenic hilum was hemostatic and the bag containing the spleen was parked above the liver. The case was then turned over to the gynecologist for the hysterectomy.

**Fig. 1:**
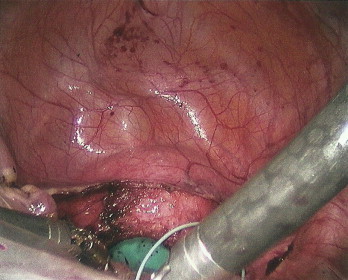
The Endo Catch bag containing the spleen was then transferred to the pelvis and retrieved with a sponge stick transvaginally. The septa bulb was replaced and the vaginal cuff was closed laparoscopically (Fig. 2).

**Fig. 2:**
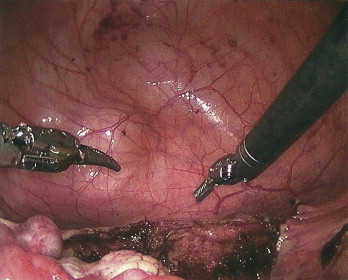
Trocar sites bigger than 5 mm were closed with a Carter Thomason (Cooper Surgical Inc^®^, Trumbull, CT) endofascial closure device. Patient was transferred to the floor post-operatively and she was started on clears later that night.

The vagina was cleansed and a Rumi II (Cooper Surgical Inc^®^, Trumbull, CT) uterine manipulator was introduced. The left lateral umbilical, sub-xiphoid and left anterior axillary line ports were reused for placement of the robotic instrument trocars. Laparoscopic transvaginal hysterectomy was performed in the standard fashion with no complications. The right adnexae were conserved. The colpotomy incision was extended till complete and the uterus was flipped forwards and amputated. The uterus was then removed via an Endo Catch bag placed through the vagina and a septal bulb on a sponge stick was placed to tent up the vaginal cuff to maintain pneumoperitoneum (Figs. 1 and 2).

Total operative time was 245 min and estimated blood loss was 50 mls.

Patient had an uneventful post-operative course and was discharged home on post-operative day 2.

Pathology showed a benign fibrous lesion in the spleen measuring 4.7 cm × 4.0 cm × 3.1 cm and this was called to represent either a sclerotic splenic hamartoma or a burned out inflammatory pseudotumor. The spleen weighed 175gms and measured 10.5 cm × 7.8 cm × 5.1 cm. The uterus weighed 210 gm with multifocal adenomyosis as well as intramural leiomyoma.

Her first post-operative clinic visit was in March and she was doing well, back to her regular physical activities. Abdominal incisions were well healed. She resumed sexual activity at 6 weeks post procedure and noted no dyspareunia or any abnormal change in sensation. She was seen in June where she noted an episode of post-coital bleeding. Physical exam revealed some raw granulation tissue at the colpotomy incision site which was managed with topical creams and postponement of sexual activity for 2 weeks. Since then, no recurrence of her vaginal bleeding was noted after resumption of coitus. To date, she remains well with no symptoms related to her surgery.

## 3 Discussion

Given the patient's history of prior vaginal deliveries, this placed her in the demographic of women who would have been more willing to accept a transvaginal procedure than a younger or nulliparous female.^11,12^ Prior pelvic surgery, which included an open oophorectomy and a subsequent completion salphingectomy for ectopic pregnancy, didn’t cause any technical difficulties in the pelvic portion of the case. There were some adhesions from the previous nephrectomy and Pfannenstiel incisions, which were easily taken down laparoscopically.

Studies have shown that although the fundus of the vagina measures only 3–4 cm across, specimens measuring more than 7 cm in width or weighing close to 500 gm may be easily removed,^13^ conceivably due to the inherent pliability of the vaginal tissues We did not experience any difficulty with specimen retrieval for either the uterus or spleen – time from completion of the circumferential colpotomy to retrieval of both organs took less than 5 min.

Microbiological contamination during transvaginal and transgastric approaches have been studied in animals^14^ and the TV route was shown to be safer with less intra-abdominal infections. A prospective NOTES registry looked at 488 patients who had undergone TV cholecystectomy.^15^ Infective complications included UTI, pouch of Douglas abscesses, vaginal mycosis and bacterial vaginitis with a combined incidence of 1%, which is similar to infection rates with conventional laparoscopy. Our patient didn’t experience any infectious complications with vaginal access or at her port sites at 18 months post-procedure.

Sexual function and fertility following vaginal access procedures remain a concern and while the fertility question remains unanswered, there appears to be no difference in sexual function.^16,17^ Our patient resumed sexual activity at 6 weeks post-operatively as advised by her gynecologist and found no difference in her sexual function. This remains a subjective and sensitive topic and there remains to be a good validating tool to broadly measure sexual function for women.

The ‘team skill set’ concept was highlighted by Rattner in his discourse on the original NOTES white paper and served as a reminder that we bring unique but complementary skills to the operating table. This was evident in this case where an urologist was involved in stenting the left ureter, a general surgeon performed the splenectomy and a gynecologist did the hysterectomy and retrieved both specimens. Cooperation was especially valuable in determining optimal sites for port placement, which allowed sharing of 3 out of the 4 initial ports.

Platforms that will blend the flexibility of an endoscope yet provide the stability to perform tissue retraction and manipulation are still in development. ‘Hybrid’ approaches have frequently necessitated the use of an additional port or two for mainly retraction and also control of insufflation, both of which are fundamental components of laparoscopic surgery but pose major stumbling blocks to progressing with our current endoscopic technology. Often, a laparoscopic camera is introduced to ‘clarify the view’ obtained with an endoscope. Thus when it is clear that the instrumentation and optics are inferior in current endoscopes compared to laparoscopes, the rationale for placing endoscopes into the peritoneal cavity is still at present, debatable.

The biggest advantage of NOSE may be that it enables mini-laparoscopy. Mini-laparoscopy refers to instruments smaller than 5 mm and can be as small as 3 mm or less. The risk of herniation looms with any abdominal incision when the fascia is breached, however by limiting the size of the trocar to 5 mm or less, the risk of herniation is close to zero and decreasing the size of the trocar incisions, even by a seemingly insignificant few millimeters, also results in decreased post-operative pain.^18^ Additionally, herniation through a vaginal colpotomy has only been described in rare case reports following vaginal hysterectomy.^19^ Thus the next logical step, at least for my institution, would be to perform a laparoscopic procedure with mini-laparoscopy and transvaginal specimen extraction, which combines the benefits of laparoscopy and transvaginal specimen retrieval.

## 4 Conclusion

The advantages of organ extraction through the transvaginal route are clear and NOSE could represent a starting point for NOTES as technologies and flexible endoscopic platforms continue to develop.

For surgeons in a community or district hospital wishing to explore transvaginal organ extraction, synchronizing the surgery with a gynecological procedure where a colpotomy would be performed regardless, would be a good starting point to explore the utility and benefits of NOSE.

## Conflict of interest statement

None.

## Funding

None.

## Ethical approval

Written informed consent was obtained from the patient for publication of this case report and the accompanying images.

## Author contributions

Jon King and Lisa Jaacks performed the surgery and assisted with procurement of the intra-operative images. They both also assisted with review of the manuscript and providing references
